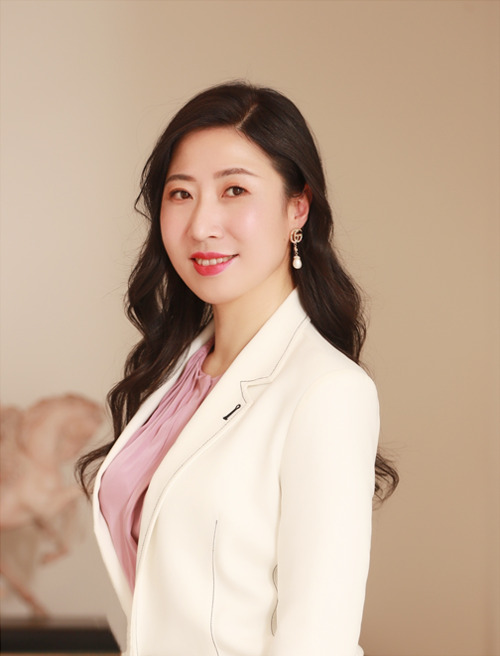# Light People: Professor Donna Strickland

**DOI:** 10.1038/s41377-021-00502-z

**Published:** 2021-03-31

**Authors:** Hui Wang

**Affiliations:** Department of International Cooperation, Changchun Institute of Optics, Fine Mechanics and Physics, Chinese Academy of Sciences, 3888 Dong Nan Hu Road, Changchun, 130033 China

**Keywords:** Lasers, LEDs and light sources, Optical physics

## Abstract

It is rare for an associate professor to win the Nobel Prize for Physics, even more so when she was only the third woman in history to do so, but Dr. Donna Strickland did just that. A permanent smile on her face expressing her friendliness, the fast speaking speed showing her quick thinking, Dr. Strickland is not only a good tutor with strong hands-on ability and high working efficiency in the eyes of her students, but also an optimistic and diligent scholar in the eyes of her peers. Taking an active part in academic societies and the popularization of science, she regards scientific research as “fun”. The famous Canadian physicist Donna Strickland talks exclusively to *Light: Science and Applications* (light).


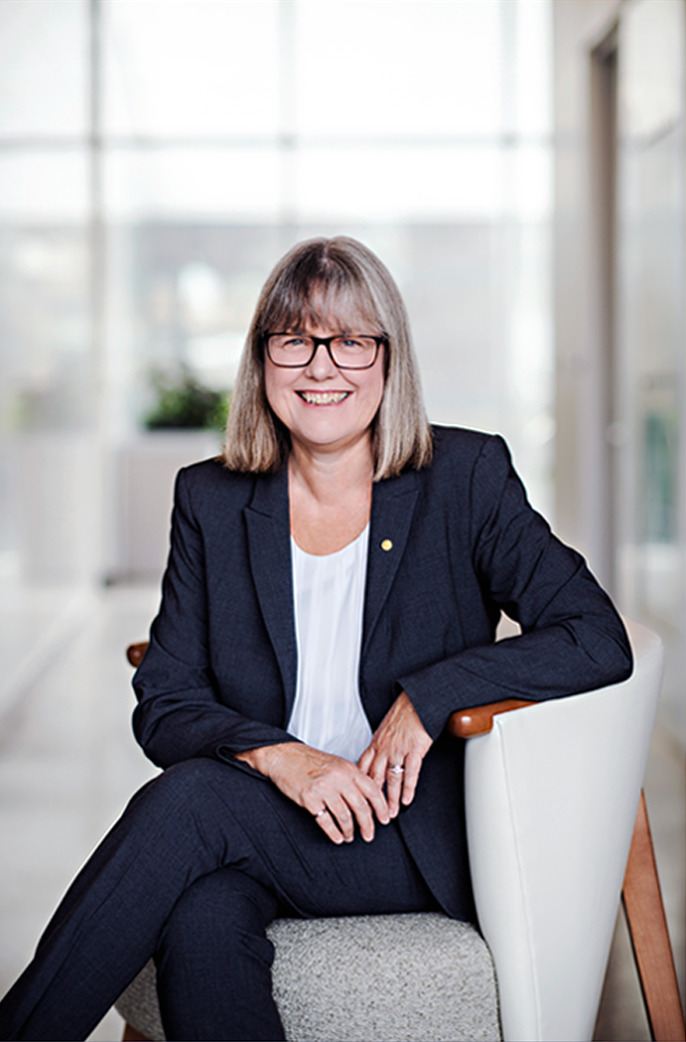


**Biography**: Donna Strickland is a professor in the Department of Physics and Astronomy at the University of Waterloo and one of the recipients of the Nobel Prize in Physics 2018 for developing chirped pulse amplification with Gérard Mourou, her PhD supervisor. Together they paved the way toward the most intense laser pulses ever created. Their research has several applications today in industry and medicine—including the cutting of a patient’s cornea in laser eye surgery and the machining of small glass parts for use in cell phones.

Strickland was a research associate at the National Research Council Canada, a physicist at Lawrence Livermore National Laboratory, and a member of technical staff at Princeton University. She is a recipient of a Sloan Research Fellowship, a Premier’s Research Excellence Award, and a Cottrell Scholar Award. She served as the president of the Optical Society (OSA) in 2013 and is a fellow of OSA, the Royal Society of Canada, and SPIE (International Society for Optics and Photonics). Strickland is an honorary fellow of the Canadian Academy of Engineering as well as the Institute of Physics. She received the Golden Plate Award from the Academy of Achievement and holds numerous honorary doctorates.

**1.** **The Chirped Pulse Amplification (CPA) technology has revolutionized the development of laser intensity, which has increased by 6–7 orders of magnitude in less than 10 years. In your opinion, is there anything that might restrict the further rapid development of CPA?**

The first few orders of magnitude were the easy ones as kilojoule level lasers already existed, but they could only amplify nanosecond long pulses. With the development of CPA along with the development of large gratings with a high damage threshold, kJ lasers could amplify picosecond pulses. Terawatt level lasers became petawatt level lasers. It has taken another 25 years to go one more magnitude and reach the 10 PW level. The power can still be increased but the progress might not be rapid. Raising the energy is costly as it requires larger-scale lasers. Using OPCPA, the pulse duration has been reduced to only a few periods of oscillation, so it won’t be easy to reduce the pulse duration further at the same wavelengths. We should be able to increase the power of CPA or OPCPA systems by another order of magnitude. China already has plans to develop a 100 PW system. Increasing the intensity by orders of magnitude will probably require reducing the wavelength. The minimum focused beam diameter is limited to wavelength dimensions as is the shortest pulse duration. For each order of magnitude reduction in wavelength, the focused intensity can then be increased by three orders of magnitude. For this to happen, we need to either develop broadband laser amplifiers that can handle the shorter wavelengths or we have to be able to convert the near infrared pulses from conventional CPA systems to the ultraviolet with very good efficiency.

**2. I have learned that your group is also focusing on the research of fiber CPA of mid-infrared generation, what are the future potential applications of fiber CPA?**

The role of fiber CPA is in applications requiring high average power as well as high peak power. This would apply to laser machining applications as well as sensing applications where the higher average power would lead to better signal to noise.

**3. Would high power attosecond laser be another important topic for CPA? If yes, what might be the main challenge?**

Attosecond pulses are necessarily out in the EUV region of the spectrum, where the period of oscillation is in the attosecond regime. As I have already answered this will most likely be what is needed to gain orders of magnitude higher intensity. We would need to combine attosecond technology with EUV and X-ray laser amplification technology. The gain medium will have to have an extremely wide gain bandwidth to maintain the attosecond duration after amplification and as far as I know, this has not yet been achieved. It is not clear whether stretching the pulse would be necessary as the gain media will most likely be a plasma and to achieve significant gain could require guiding in a plasma channel and so self-focusing may not be an issue. It could be similar to fiber CPA where the beam is confined in a channel, but nonlinearities would help increase the bandwidth and cancel gain narrowing.

**4. How do you think the femtosecond laser fabrication, perhaps one of the most important applications of the CPA technology, has led to the advancement of fabrication, especially for micro/nano fabrication of special materials?**

Long pulse or continuous wave high power laser machining is a result of a thermal process through absorption of the light. This has two consequences, first, the spot diameter is affected by thermal migration, and secondly, the machining must start at the surface where the light is initially absorbed. With CPA, the machining is initiated by nonlinear ionization which occurs only in the focal region. The light does not need to be absorbed so it can machine transparent objects such as glass and the cornea of an eye. Also, the beam focus can occur inside the medium rather than on the surface allowing micro-modifications to the interior of the material. As an example, the refractive index can be changed and so waveguides can be written inside the glass.

**5. What difficulties did you encounter in your research on CPA? How did you overcome them?**

I don’t remember too many difficulties. We damaged laser rods and had to wait for replacements. The lasers were old and so they quit working often. Luckily, we had a very good electronics person working with us and he was able to keep the lasers working. It was not my responsibility. The one time I remember getting unexpected results was when we first amplified the chirped pulse and looked at its spectrum. There was an oscillation in the signal that should not have been there based simply on the gain spectrum. It didn’t take us too long to realize that the oscillation looked like it was caused by a 1 mm glass etalon. The quarter waveplate that was used along with a Pockel cell for switching the pulses in and out of the regenerative amplifier was indeed a 1 mm piece of glass. We had used the same combination of waveplate and Pockel cell in regenerative amplifiers for narrow spectra and had no problems because the oscillation period was broader than that spectrum. We realized the solution for CPA was to rotate the Pockel cell so that in its off position, it would act as a quarter waveplate and we could then remove the 1 mm quarter waveplate.

**6. You have mentioned that when your Ph.D. supervisor, Dr. Gerard Mourou, said the CPA technique would make petawatt lasers possible, you thought that his remark was audacious, but he was correct. You also once said that the idea (CPA) was Mourou’s, but you got to make it work. Do you think it is the ideal student-teacher relationship that the senior scientists should guide the young researchers by coming up with original ideas and showing them the research prospects, and the young should have the capability of understanding and turning the ideas into reality? How do you mentor your students?**

There are probably many ways for a supervisor and student to work together. New science is a result usually of an exchange of ideas. When students consider joining my group, we have a discussion of the ongoing projects. Since it is an experimental group, initially the new student’s project will be limited to working with existing equipment. It is through many discussions together that we agree on what would be best to study for their thesis to work toward overall goals in the group.

**7. You were not a “full” professor when you won the Nobel Prize in Physics because you had never applied for that rank, even though you were already an excellent and celebrated scientist, for instance, you are a former president of The Optical Society (OSA) and director of Academic Affairs for the Canadian Association of Physicists. So what’s the reason that you did not apply for full professorship?**

The title Full Professor means different things at different institutions. In my case, I simply didn’t see sufficient benefit to being a Full Professor. As an Assistant Professor, you must apply for tenure and application to Associate Professor. It is your own decision to apply to become a Full Professor. I want to be clear that it was not because I was a woman. Except for me, all the female faculty in the department, with sufficient experience were Full Professors at the time. I had male colleagues suggest to me that I apply to become a Full Professor.

**8. As the OSA’s Vice President you visited the Changchun Institute of Optics, Fine Mechanics and Physics (CIOMP), Chinese Academy of Sciences, with an OSA delegation in 2012 when CIOMP celebrated its 60**^**th**^ **birthday. What’s your impression of CIOMP?**

I was very happy to be included as part of the OSA delegation at the 60^th^ birthday of CIOMP. CIOMP is known as the “cradle of China’s optics” and the celebration showcased the wonderful advancements that the institute has made over the 60 years. We were shown state-of-the-art research facilities and the tours were given by very knowledgeable and professional students. OSA’s partnership with this well-known institute has provided opportunities for members of our global community to interact and foster collaboration. In fact, OSA hosted IONS-Asia 3/Changchun at CIOMP, just ahead of CIOMP’s 60^th^-anniversary celebration. OSA was then proud to partner with the Institute to host the second CIOMP-OSA Summer Session in 2013. OSA was honored to invite CIOMP to participate in OSA’s 100^th^-anniversary celebration which took place in 2016.


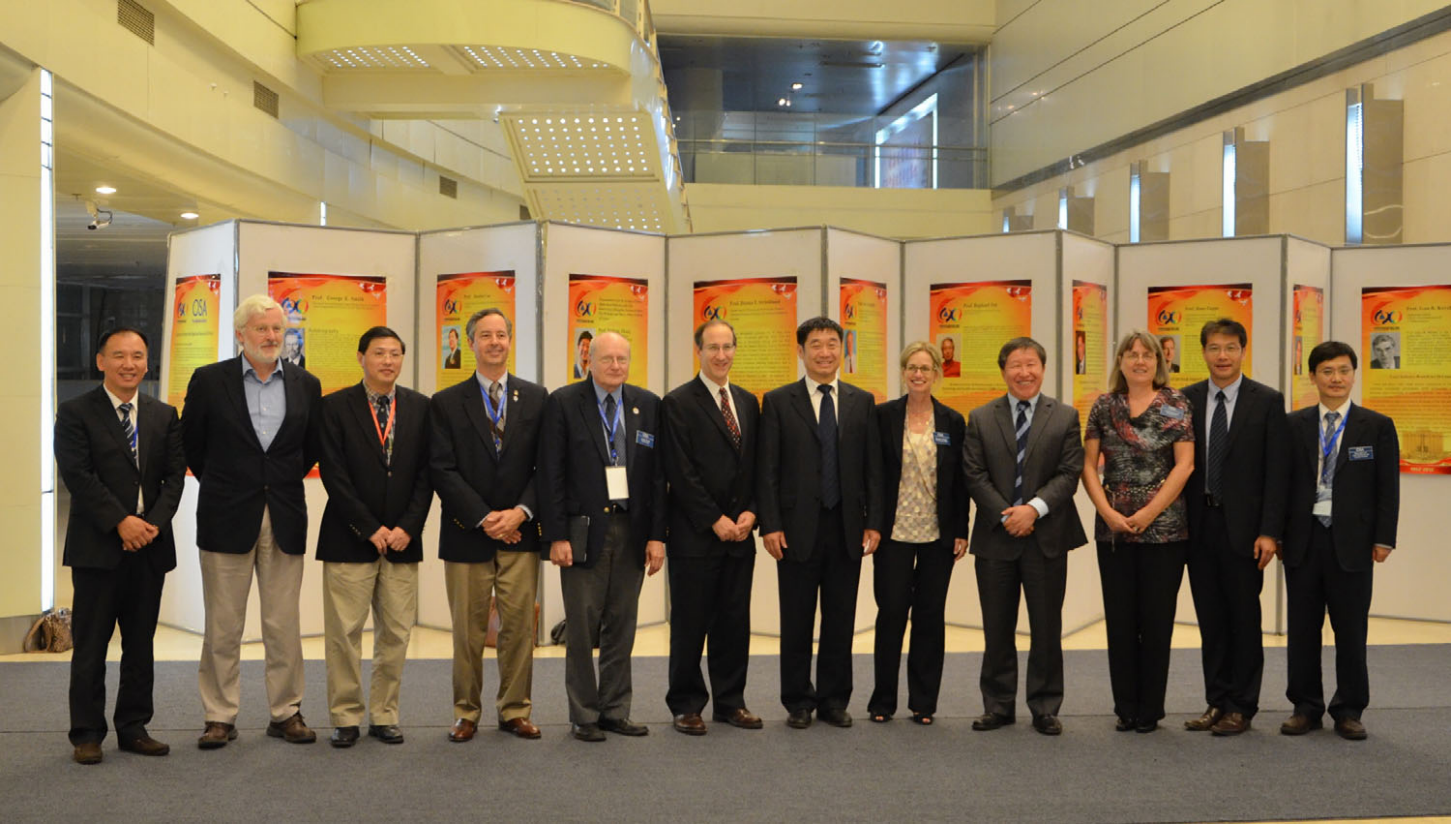
**Fig. 1** Group photo of CIOMP executives and the OSA delegation at CIOMP in 2012.


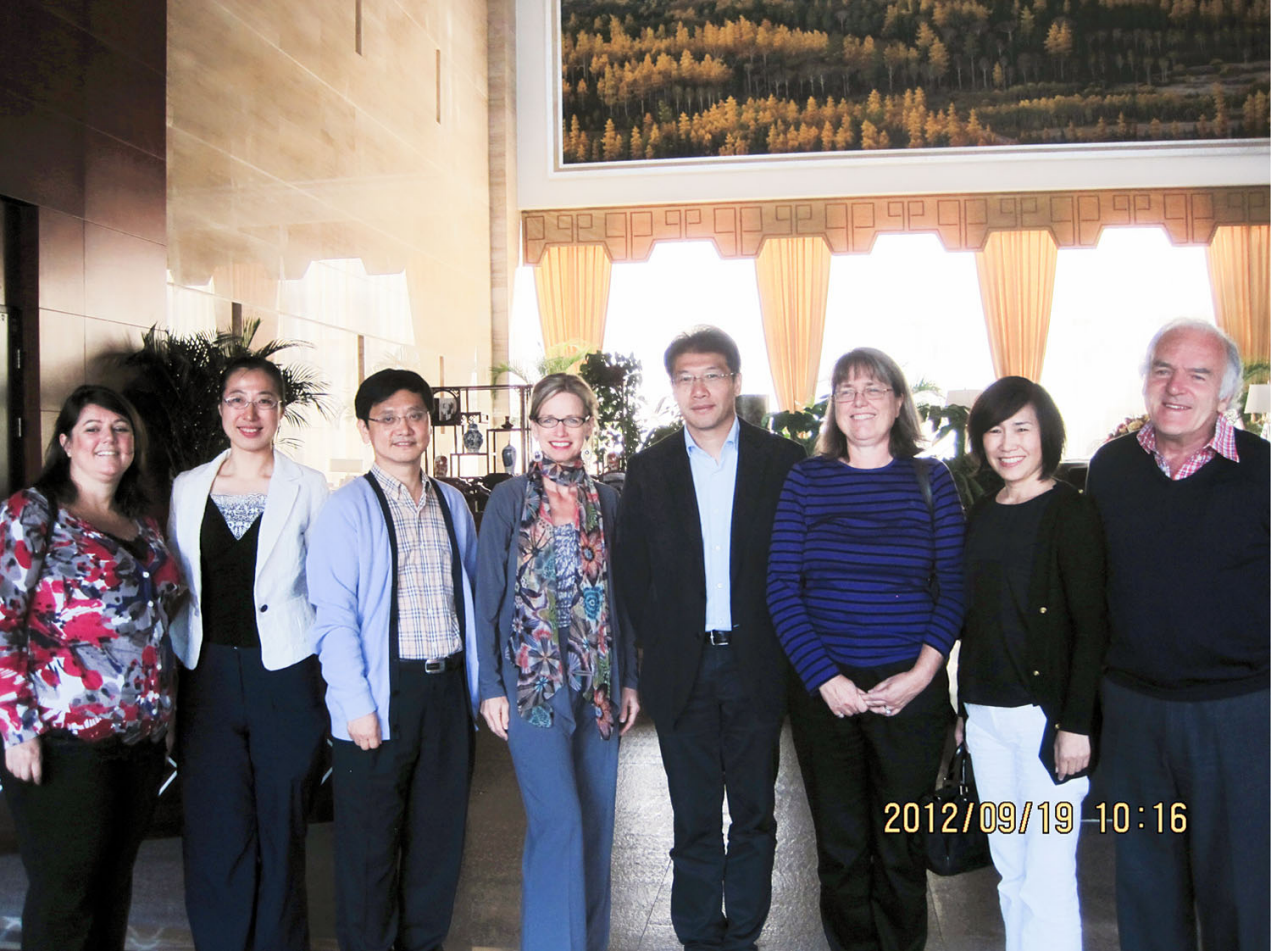
**Fig. 2** Dr. Strickland with some founding members of the journal *Light: Science & Applications* and her OSA colleagues.


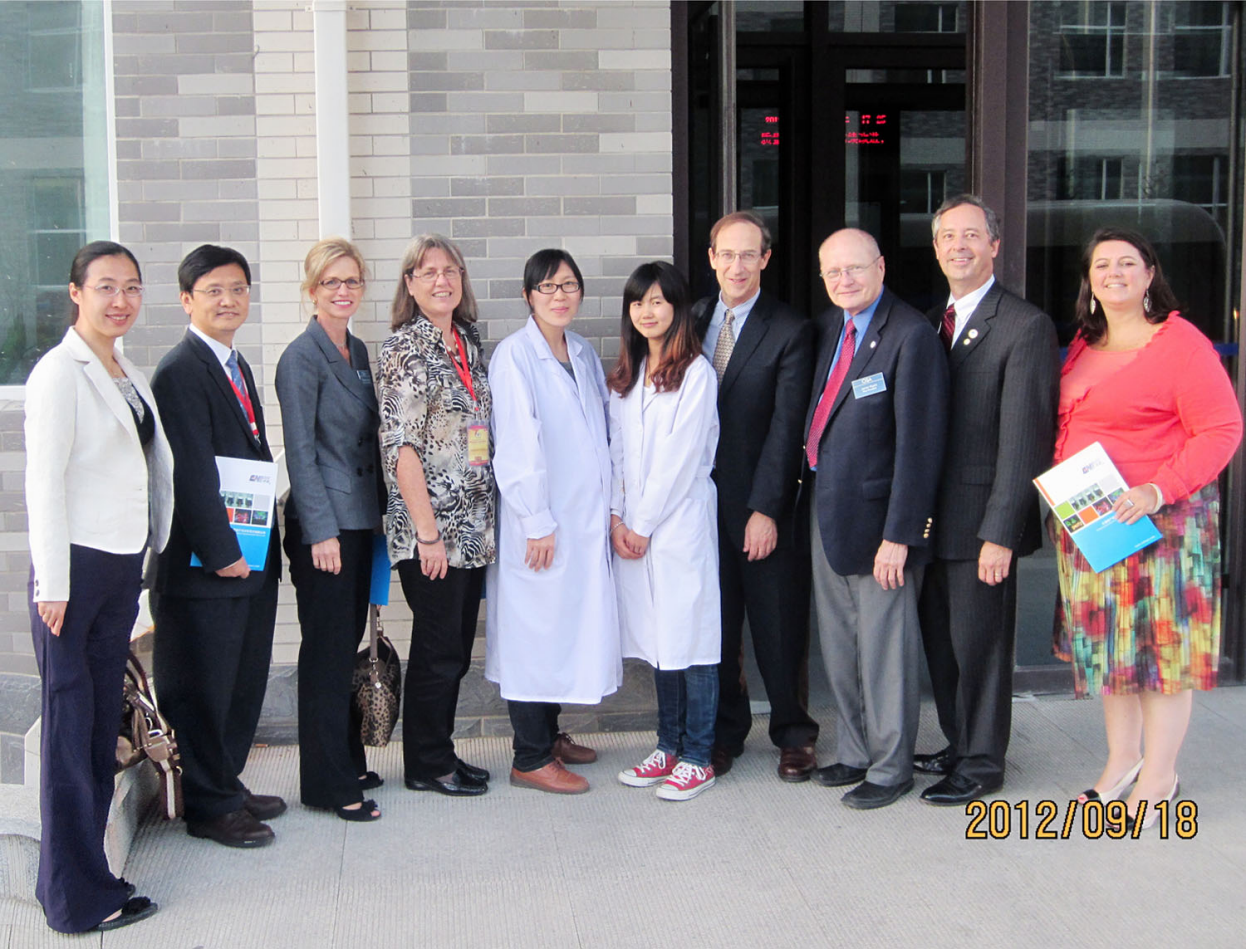
**Fig. 3** The OSA delegation visits the Changchun New Industries Optoelectronics Technology Co., Ltd. based at CIOMP.

**9. What kind of mentality and quality should scientific researchers have? Should we stick with a research topic if its results continuously fall short of our expectations?** **(This question is chosen from a selection of questions from the CIOMP graduate students.)**

I believe researchers need creativity to come up with new ways to investigate a scientific question. They certainly need patience because the answers to most scientific questions do not come easily. It can be difficult to know when to stop pursuing an idea. You have to stop when you cannot think of another avenue to find the solution to the problem. You will stick with problems longer if you think the answer is truly worth investigating.

**10. The COVID-19 Pandemic has delayed or interrupted the research work of many academics. Most hard hit are young scholars who are at the start of their careers. What suggestions or advice would you give to these young people concerning their future careers?** **(This question is chosen from a selection of questions from the CIOMP graduate students.)**

I agree that Covid has put added stress on young researchers who need to show strong research output to get the next position. It is probably harder on the experimentalists than theorists because they not only have lost time in the lab doing the needed experiments, but there have been delays getting new equipment and servicing of equipment is being slowed because of travel restrictions. For all scientists, networking has become more difficult as conferences cannot be held in person. I am sure that senior researchers and managers are very aware of the situation and will take Covid into account when reviewing the output of scientists. My advice would be to work on what you can accomplish during these times. Look for new ways to network with colleagues in your field. Most importantly try your best to stay positive.

**11. How do you choose your research topic? According to your interest or for its future application? Why?**

I choose based on interest mostly. Sometimes it is an opportunity. Sometimes the topic is brought to me. My group developed a two-color CPA laser system because I inherited a two-color, short-pulse laser. I wondered what I would do with this laser and of course, I would CPA it. That led me to look into what nonlinear optics experiments needed two colors. When the group had built the system, Dr. Leonid Losev who was working in Russia asked if he could come to Waterloo to use the laser to try out his idea to increase the efficiency of a nonlinear optical effect known as multi-frequency Raman generation. That is how I started studying multi-frequency Raman generation. Then my graduate student noticed that there were extra peaks between the multiple Raman peaks and together we wondered what they were so the group started investigating these extra peaks just because we wanted to know the answer. I am also championing the Global Environmental Measurement and Monitoring initiative with OSA. Since I also work on mid-infrared generation I will look to see if it can be applied to environmental sensing. I am open to new avenues of research from a number of different sources.

**12. You are reputed to be a great teacher. I heard once your student accidentally broke an infrared mirror but you only said “don’t worry! It’s common in the laboratory”. Then you spent $2000 on a new one. Another story was also from a former student of yours who said that he received feedback within three days of submitting his graduation thesis to you, so his graduation was very smooth. What qualities do you think a good mentor should have?**

Well, I don’t know who you have been talking to. Thank you for finding the students who had nice things to say. I would think most experimental supervisors would understand equipment being broken as long as the student doesn’t make a habit of it. I certainly broke things along the way and my supervisors were not angry with me, so I would have no business being angry with my students. I do think it is the duty of the supervisor to provide feedback in a timely manner. I think the supervisor is there to be encouraging for the student and mostly to help the student to learn the field and help solve the scientific and technical problems and teach them how to ask the correct questions.

**13. You are a senior member or chairman of many national and international academic organizations. How has this affected your academic work?**

First, I would say that the organizations benefit researchers and so that it is in our interest to support the organizations. I have also found that volunteering with organizations gives you a much broader understanding of your field. It also helps you make connections not only with scientists outside your research field, but also with government and society in general. This broad networking opens avenues that you might not have otherwise considered.

**14. You said you have great faith in lasers, but no one’s putting one near your eye. Why is that? Is it a kind of occupational psychology?**

I am a very squeamish person. I would not have surgery unless absolutely necessary. I would simply rather wear glasses than have surgery. If I had to have surgery, then I think a laser is an excellent surgical tool.

**15. Both you and your daughter are engaged in scientific research. Do you think it is a natural love of science or family influence?**

I suppose it is some of both. Both my husband and I are scientists in the same field so our children would have grown up listening to us discuss our work. She would be more aware of science as a career. She was also a good math and science student and inherently inquisitive, so it was also a natural choice for her.

**16. You once said in an interview that you were raised by a strong woman who would follow her heart not listening to anybody else. How do you rate this quality?**

I think we need confidence in ourselves and I think my mother helped instill confidence in me. I don’t mean that you should not listen to other people’s points of view, but when it comes to making decisions about your own life, you know better than anyone what you want to do.

**17. Have you heard of the journal** ***Light: Science & Applications*? What do you think of it?**

Yes of course I have heard of *Light: Science & Applications*. The journal’s contributions to the dissemination of advancements in this field of research support the scientific community.

**18. How do you choose which journals to submit your articles to?**

As you have noted, I have been the president of OSA: The Optical Society, which publishes 19 high-quality, peer-reviewed journals in its publishing portfolio, which also serve the full breadth of the optics and photonics community. I will always be partial to OSA journals, however, I usually let the students decide where they want to submit their papers.


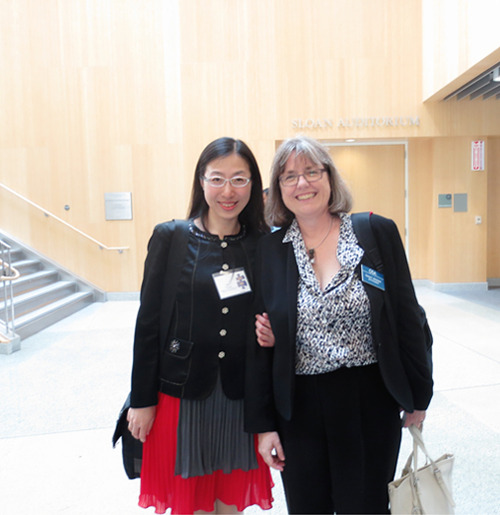
**Fig. 4** Prof. Strickland, then OSA President with Ms. Hui Wang, light special correspondent for this interview and one of the journal’s founding members, at an international conference at the University of Rochester in 2013.

**Light** **special correspondent**

*Hui Wang is the Deputy Director of the Office of International Cooperation in the Changchun Institute of Optics, Fine Mechanics and Physics (CIOMP), Chinese Academy of Sciences (CAS). She currently works on international communication and cooperation for the CIOMP and was a founding member for the Nature Publishing Group and CIOMP joint journal* *Light: Science & Applications*. *She is the founder of “Rose in Science” and has published several articles in* *Acta Editologica*, *International Talent*, *etc., and was invited to take an interview by SPIE Women in Optics, which was published in 2015*.